# A novel composite conductive microfiltration membrane and its anti-fouling performance with an external electric field in membrane bioreactors

**DOI:** 10.1038/srep09268

**Published:** 2015-03-18

**Authors:** Jian Huang, Zhiwei Wang, Junyao Zhang, Xingran Zhang, Jinxing Ma, Zhichao Wu

**Affiliations:** 1 State Key Laboratory of Pollution Control and Resource Reuse, College of Environmental Science and Engineering, Tongji University, Siping Road 1239, Shanghai 200092, P.R. China

## Abstract

Membrane fouling remains an obstacle to wide-spread applications of membrane bioreactors (MBRs) for wastewater treatment and reclamation. Herein, we report a simple method to prepare a composite conductive microfiltration (MF) membrane by introducing a stainless steel mesh into a polymeric MF membrane and to effectively control its fouling by applying an external electric field. Linear sweep voltammetry and electrochemical impedance spectroscopy analyses showed that this conductive membrane had very good electrochemical properties. Batch tests demonstrated its anti-fouling ability in filtration of bovine serum albumin, sodium alginate, humic acid and silicon dioxide particles as model foulants. The fouling rate in continuous-flow MBRs treating wastewater was also decreased by about 50% for this conductive membrane with 2 V/cm electric field compared to the control test during long-term operation. The enhanced electrostatic repulsive force between foulants and membrane, *in-situ* cleaning by H_2_O_2_ generated from oxygen reduction, and decreased production of soluble microbial products and extracellular polymeric substances contributed to fouling mitigation in this MBR. The results of this study shed light on the control strategy of membrane fouling for achieving a sustainable operation of MBRs.

Membrane bioreactors (MBRs), which integrate conventional activated sludge process with membrane separation, have been widely used for both industrial and municipal wastewater treatment^1^,^2^,^3^. Nevertheless, membrane fouling is an inevitable problem and remains one of major obstacles to wide-spread applications^2^,^4^. Membrane fouling causes flux decline or trans-membrane pressure increase, leading to frequent membrane cleaning and membrane replacement. In general, membrane fouling is attributed to deposition/adsorption of particulate and soluble materials on membrane surfaces and/or into membrane pores. Membrane modification, operating parameters optimization, and mixed liquor filterability improvement are widely-used three approaches to suppress membrane fouling in MBRs^5^,^6^.

Since most of membrane foulants including sludge flocs, soluble microbial products (SMP) and extracellular polymeric substances (EPS) are generally negatively charged, it may be possible to mitigate membrane fouling by increasing electrostatic repulsion between membranes and foulants. Recently, applying an external electric field for membrane fouling suppression has received much attention among research communities. Akamatsu et al.^2^ developed a membrane filtration cell by putting a microfiltration (MF) membrane between a pair of electrodes made of platinum; they observed that the enhanced electric repulsive force can facilitate removing sludge flocs away from membranes in the presence of an electric field provided by a DC power. Similar results were observed by Liu et al. using similar membrane configuration in an MBR, and 20–25% flux enhancement was achieved^7^. However, in these researches, cathodes were placed around/near membrane to induce an electric repulsive force around the membrane, which thus may impair the efficiency of electric field and impact its anti-fouling performance.

In order to efficiently utilize the electric repulsive force, using conductive membranes to directly serve as cathodes has been further proposed. Professor Jassyby and coworkers developed conductive carbon nanotube-polymer composite membranes for ultrafiltration, nanofiltration and reverse osmosis processes^8^,^9^,^10^. They observed about 33% and 51% decreases in operating pressure while applying −3 V and −5 V during 100 min batch filtration of alginic acid^10^. For MBR applications, Liu et al. modified a polyester cathode membrane by coating graphene/polypyrrole, and by applying 1 V/cm electric field, an increase of 20% in permeate volume was obtained^11^. Stainless steel mesh was also used as conductive membrane (cathode) in MBRs^12^,^13^. These research efforts in MBRs provide useful information to improve the efficiency of electric field in mitigating membrane fouling. However, among these MBR studies, the used membranes had large pore sizes, which are also termed dynamic membranes^14^,^15^. In general, dynamic membranes, compared to MF membranes, have lower membrane fouling rate since separation is carried out by the dynamic membrane layer formed by large particles^16^,^17^. Over-growth dynamic membrane layer can be controlled by enhancing hydraulic conditions since the layer is self-forming and reversible^18^. Currently, MF membranes are the predominant membrane types used in MBRs, and membrane fouling mitigation for MF membranes is much more urgent as MF fouling is generally more complicated and difficult to control compared to dynamic membranes^16^,^17^,^19^. However, to date, information on developing conductive MF membranes and mitigating their fouling by applying an electric field in MBRs is very scarce. Conductive carbon nanotube-polymer composite membranes^8^,^9^,^10^ have not been applied in MBRs.

In this study we report a novel composite conductive MF membrane by introducing a stainless steel mesh between the supporting layer and active layer of a polymeric MF membrane without changing its surface physicochemical properties. The prepared conductive MF membrane can be directly used as not only a cathode but a separation membrane. Anti-fouling performance of this conductive MF membrane with a 2 V/cm external electric field was evaluated in batch tests using model foulants and also in continuous-flow MBRs.

## Results

### Membrane properties

**Figure 1:**
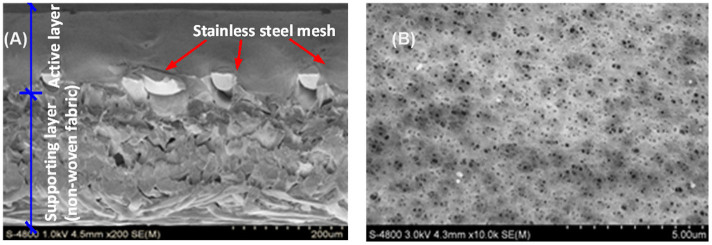
Representative SEM images of the conductive membrane. (A) Cross-section morphology, and (B) surface morphology.

Surface and cross-sectional scanning electron microscope (SEM) images of the conductive membrane are shown in Fig. 1. As shown in Fig. 1 A, the stainless steel mesh was well embedded in the active layer due to the strong adhesive of the casting membrane solution. The membrane surface exhibited evenly distributed micropores with an average value of 0.062 ± 0.024 μm (see Fig. 1 B). The conductive MF membrane properties including pure water flux (PWF), contact angle (CA), roughness and pore size are summarized in Table S1. There is no obvious change in membrane physicochemical properties compared to original polyvinylidene fluoride (PVDF) membranes. However, other conductive membrane preparation strategies, e.g., carbon nanotube-polymer composite membrane^8^,^9^,^10^ and graphene/polypyrrole-coated membrane^11^, can result in changes in membrane surface properties compared to pristine membranes. The tensile strength for the conductive MF membrane and the control membrane (without stainless steel) in our study was 38.6 ± 1.5 MPa (*n* = 5) and 33.4 ± 0.9 MPa (*n* = 5), respectively, suggesting that the physical strength was improved with the introduction of stainless steel mesh into the membrane.

**Figure 2:**
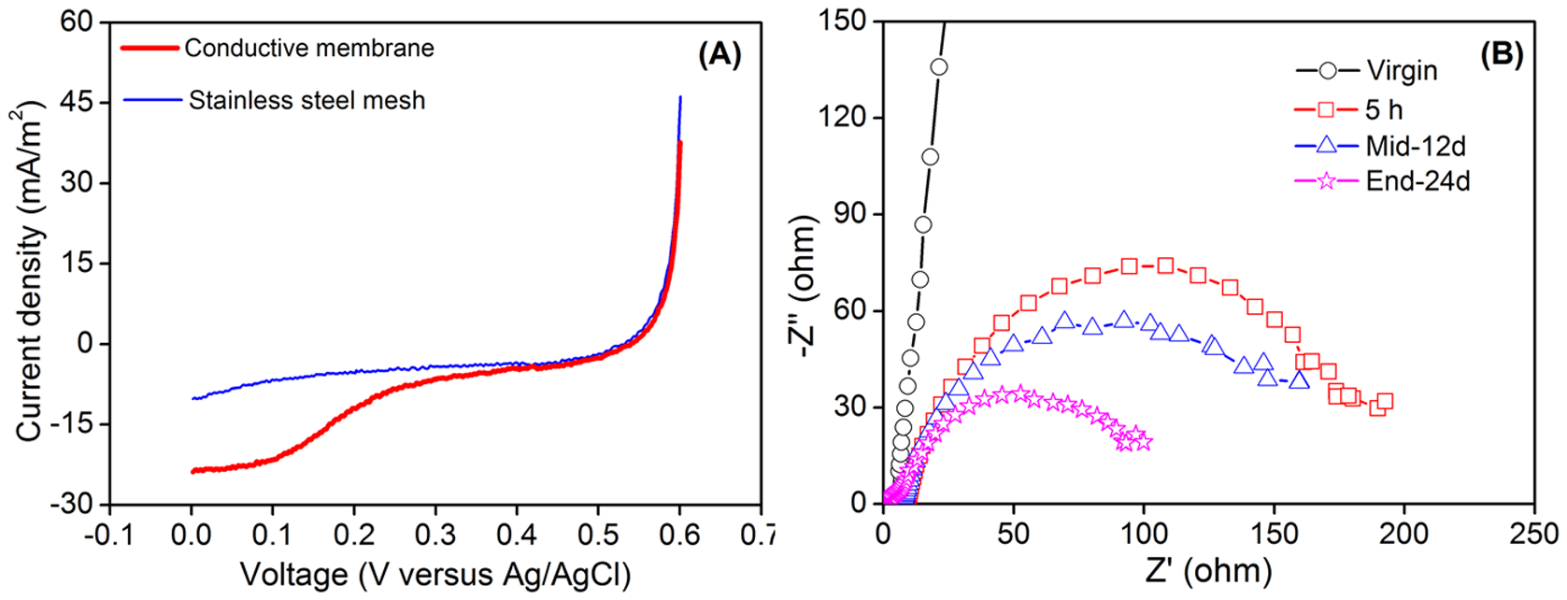
(A) Linear sweep voltammetry (LSV) of conductive MF membrane and stainless steel mesh, and (B) Nyquist plots of electrochemical impedance spectroscopy (EIS) spectra of the conductive MF membrane under practical operation.

The composite conductive MF membrane and the stainless steel mesh used in the membrane preparation were examined using linear sweep voltammetry (LSV) to evaluate the effect of casting membrane solution on electrochemical performance. Fig. 2(A) demonstrates that this conductive MF membrane had similar current densities at higher scanned potentials (>0.4 V) to the stainless steel mesh, while the conductive MF membrane performed better at lower potentials (<0.4 V). Higher current response (absolute value) of this conductive membrane at lower potentials might be attributed to the hydrophilicity of the casting membrane solution. Water contact angle was 69.9° ± 3.2 and 103.2° ± 1.34 for this conductive membrane and stainless steel mesh, respectively. Low electrolyte affinities at the interface between electrolyte and cathode surface can lead to insufficient ion transport, i.e., an ion transport resistance, while enhanced hydrophilicity increases the accessibility of electrolyte and thus improves ion transport^20^,^21^. This demonstrates that covering membrane polymer on stainless steel mesh for making conductive MF membrane did not negatively affect the inherent electrochemical properties of the stainless steel mesh.

In order to further evaluate the electrochemical performance of the conductive MF membrane during operation in a continuous-flow MBR (electrochemical MBR), electrochemical impedance spectroscopy (EIS) was conducted and the results are shown in Fig. 2(B). In Nyquist plots, the impedance at high frequency limit is defined as the ohmic resistance (*R*s) of cathode and the diameter of the semicircle is the polarization resistance (*R*p) of the cathode^22^. The appearance of *R*p could be attributed to the ionic migration process at the electrolyte and electrode interface. As shown in Fig. 2(B), *R*s of the virgin conductive MF membrane was only about 2.6 Ω and remained constant with operation time, indicating a good conductivity of this membrane. In addition, Nyquist plots displayed a straight line for the virgin conductive membrane, indicating that *R*p accounts for the majority of internal resistance when the electrochemical MBR was initiated. However, the *R*p value was notably decreased to about 200 Ω after 5-h operation and decreased to about 105 Ω after 24-d operation. The decrease of *R*p with the increase of operation time was mainly due to the catalytic capability of microbes on the cathode surface. Microbes (biofilm) on cathode surface can catalyze oxygen reduction and reduce charge transfer resistance^23^,^24^. EIS results demonstrate a good electrochemical performance of this conductive MF membrane for practical applications.

### Anti-fouling performance in filtration of model foulants

**Figure 3:**
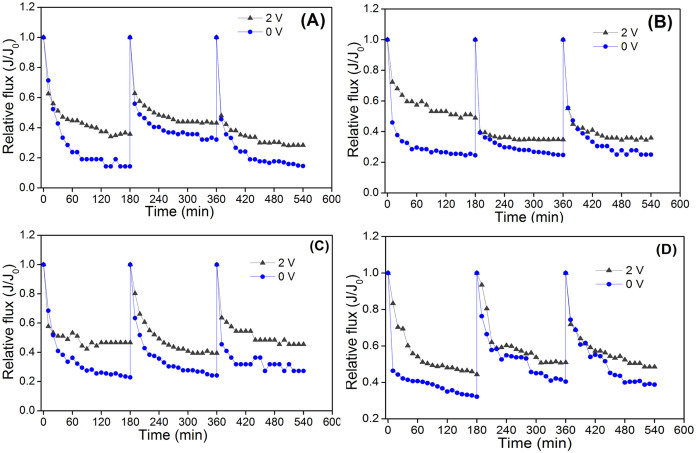
Changes of relative fluxes using (A) bovine serum albumin, (B) sodium alginate, (C) humic acid and (D) silicon dioxide particles as model foulants with and without an electric field.

Bovine serum albumin, sodium alginate, humic acid and silicon dioxide particles are regarded as typical model foulants for protein, carbohydrate, humic acid and suspended particles^25^,^26^,^27^. The filtration behaviours of this conductive membrane with electric field in the model foulant solutions can indicate their antifouling performance compared to the control test without electric field. An electrical field with the strength of 2 V/cm was applied on the membrane, and changes in the relative flux (*J*/*J*_0_) over time for the different model foulants are shown in Fig. 3. From Fig. 3, it can be observed that the conductive membrane with 2 V/cm electric field performed better, i.e., a lower decrease rate of relative fluxes (*J*/*J*_0_), compared to the control test (0 V/cm) under all investigated conditions. This demonstrates that the presence of electric field is effective in mitigating membrane fouling caused by all the model foulants. Two factors might contribute to the fouling mitigation. One is related to the repulsive force between the membrane surface and the negatively charged model foulants (see Table S2). The presence of electric field near the conductive membrane surface enhanced the repulsive force compared to the control test^2^. The other factor may be due to the generation of H_2_O_2_ at the cathode. It has been reported that H_2_O_2_ generation is observed in electrochemical systems^12^,^28^,^29^. Therefore, the produced H_2_O_2_ can be used for cleaning membrane in situ, leading to a decreased fouling potential^30^. The production of H_2_O_2_ in this study will be analyzed later.

### Pollutant removal in continuous-flow MBRs

**Figure 4:**
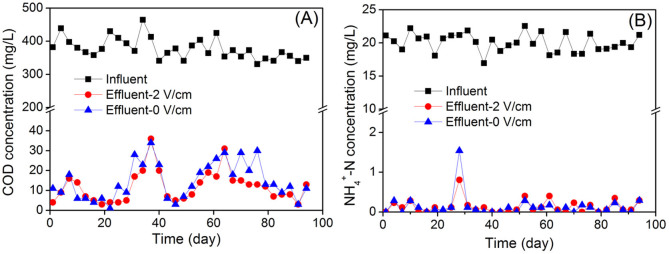
Removal of (A) COD and (B) NH_4_^+^-N in two MBR systems.

Pollutant removal efficiencies in the two parallel MBRs were monitored during long-term operation. Chemical oxygen demand (COD) and ammonium (NH_4_^+^-N) removal of two MBR systems are illustrated in Fig. 4. During 96-d operation, the average effluent COD concentration and COD removal efficiency for the control MBR were 14.7 ± 9.3 mg/L and 96.1 ± 2.5%, and 11.8 ± 7.8 mg/L and 96.9 ± 2.1% for the electrochemical MBR (with electric field), showing that the electrochemical MBR achieved slightly better permeate quality. The removal efficiencies in NH_4_^+^-N in the two reactors were almost the same (>99.3%). The results indicate that the MBR in the presence of 2 V/cm electric field also had no adverse impacts on pollutant removal.

### Membrane fouling reduction

**Figure 5:**
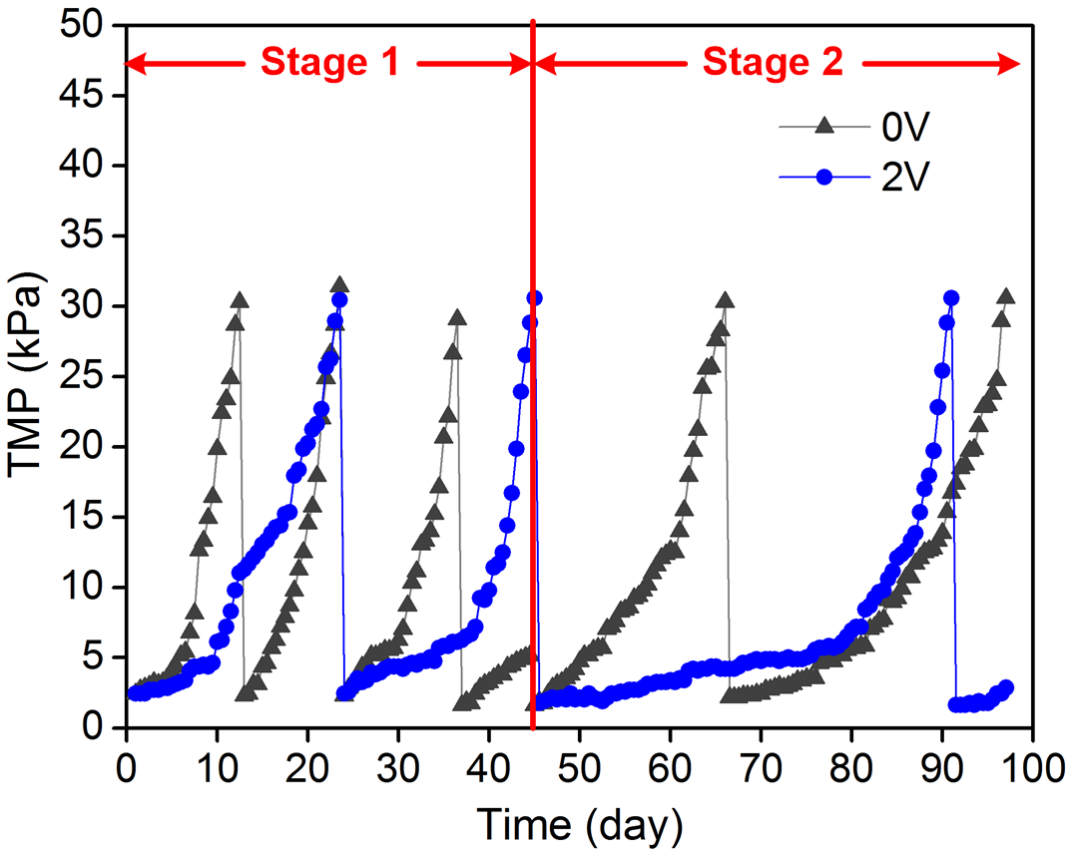
Comparison of TMP evolution between two MBR systems.

Fig. 5 illustrates the evolution of TMP in the two MBR systems. Compared with the control MBR, membrane fouling in the electrochemical MBR (with electric field) was significantly reduced during long-term operation. At stage 1 with specific aeration demand by projected membrane area (SAD_m_) 100 m^3^/(m^2^·h), the average mixed liquor suspended solids (MLSS) concentration was maintained at 6.4 g/L for the control MBR and 6.7 g/L for the electrochemical MBR. At this stage, the membrane in the electrochemical MBR was cleaned twice during 45 d operation, while the control MBR underwent three cleaning procedures. At stage 2, for the control MBR and the electrochemical MBR, the average MLSS concentrations were 6.7 g/L and 7.3 g/L, respectively. At this stage, the duration of an operation cycle for both two membranes was extended (Fig. 5). This was because that the SAD_m_ was increased from 100 m^3^/(m^2^·h) at stage 1 to 150 m^3^/(m^2^·h) at stage 2. An increase of SAD_m_ can lead to an increased cross-flow velocity (CFV) along membrane surfaces and thus an improved filtration performance^2^,^31^. A higher CFV can induce a greater shear stress along membrane surfaces, which can dislodge deposited foulants and reduce fouling^32^. Tran et al. reported that the cake resistances under shear stresses of 0.9, 2.6 and 4.9 kPa were 56, 27 and 9 × 10^11^ m^−1^, respectively^33^. It can be also observed that the operation cycle for the membrane in this electrochemical MBR was extended to 46 days, which was much longer than the membrane operated in the control MBR (25 days on average) at stage 2. The long-term performance of this electrochemical MBR again confirmed that this conductive MF membrane with electrical field could efficiently mitigate membrane fouling.

As mentioned in batch filtration of model foulants, one factor contributing to fouling reduction is the enhanced repulsive force between membrane foulants and membranes. Zeta potentials of SMP, EPS and sludge in the MBRs during the experiment are shown in Fig. S1. It is evident that SMP, EPS and sludge are all negatively charged under investigated conditions, and thus repulsive force takes effect in the presence of electric field. In addition, the generated H_2_O_2_ in the electrochemical MBR could also facilitate mitigating membrane fouling through *in-situ* membrane cleaning. In this study, about 0.95 ± 0.21 mg/L (*n* = 24) H_2_O_2_ in this electrochemical MBR was detected, whereas there was hardly any H_2_O_2_ detected in the control MBR. This is mainly attributed to the fact that H_2_O_2_ can be generated in situ at cathodes of bioelectrochemical systems through reduction of oxygen^12^,^28^,^29^.

**Figure 6:**
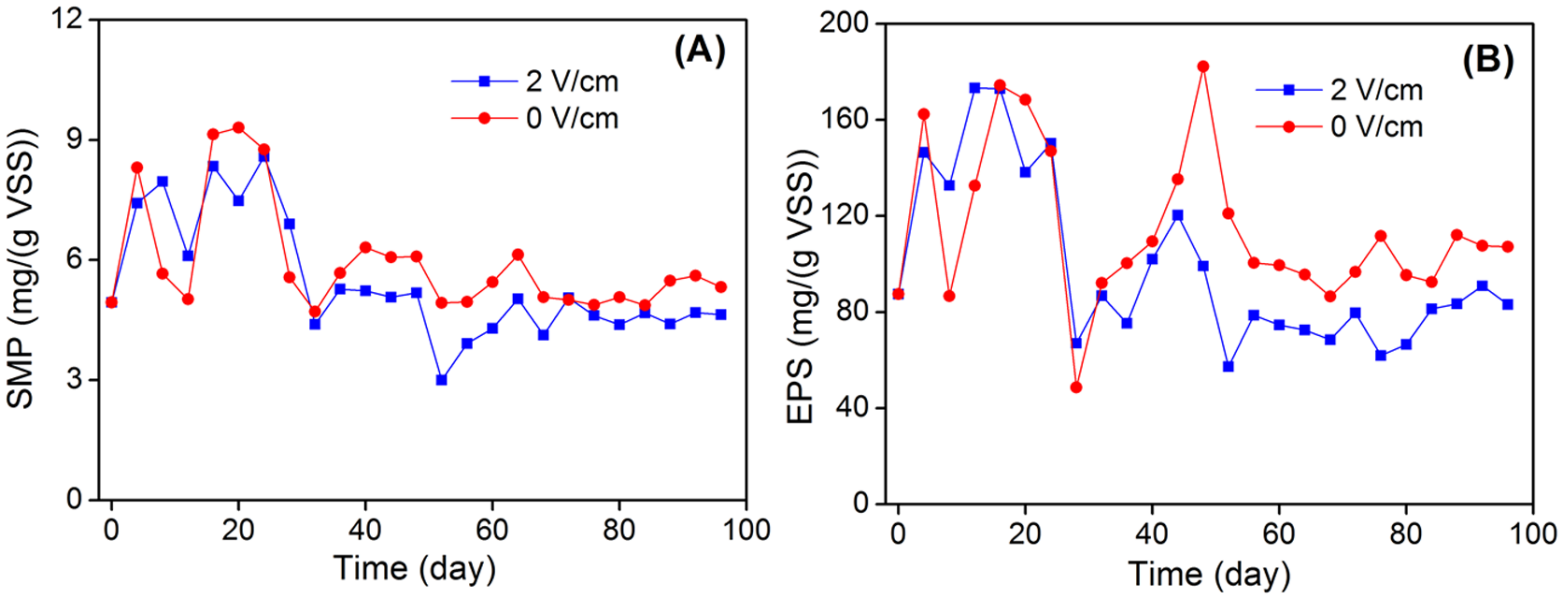
Comparison of (A) SMP and (B) EPS concentrations in the electrochemical MBR and the control MBR.

Concentrations of SMP and EPS in both MBR systems (Fig. 6) were also monitored for comparison since they are regarded as major foulants^34^. After about 24-d operation, the concentrations of SMP and EPS detected in the electrochemical MBR were lower than those of the control MBR. Higher concentrations of SMP and EPS could lead to more serious membrane fouling for the control MBR^22^,^35^. Since the two MBRs were inoculated with same sludge and operated under same conditions, the differences in SMP and EPS concentrations should be ascribed to the presence of electric field.

As mentioned above, the concentrations of EPS and SMP were impacted by the presence of an electric filed in the reactor, and it is therefore difficult to determine whether the reduced fouling was attributed to the decreased foulant concentration or the presence of the electric field. In order to further clarify this, an MBR with two membrane modules was constructed, in which one membrane module with 2 V/cm and the other without electric field were operated in parallel. In this way, the concentrations of foulants were kept identical. The TMP changes of these two membrane modules are shown in Fig. S2. As shown in Fig. S2, the operation cycle for the membrane with an electric field of 2 V/cm is much longer than the control membrane module, indicating that the reduced fouling observed on this membrane was due to the presence of the electric field.

## Discussion

**Figure 7:**
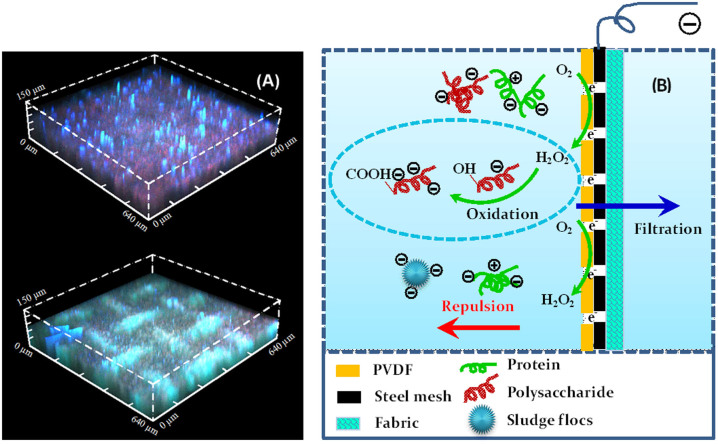
(A) CLSM images of used membranes in the control MBR (upper part) and the electrochemical MBR (lower part), and (B) schematic illustration of anti-fouling mechanisms. Symbols for figure 7A: Red for α-polysaccharides (α-mannopyranosyl, α-glucopyranosyl stained with ConA); blue for β-polysaccharides (β-D-glucopyranose stained with Calcofour white); Green for proteins (stained with FITC).

For further elucidating the anti-fouling mechanisms, confocal laser scanning microscope (CLSM) was used to visualize the membranes at the end of operation (Fig. 7A). It is evident that the electrochemical MBR had a thinner fouling layer compared to the control MBR. Furthermore, it is very interesting to find that the foulant compositions were much different. In the control MBR, polysaccharides (e.g., α-mannopyranosyl, α-glucopyranosyl and β-D-glucopyranose) accounted for a larger proportion of the deposited foulants compared to the electrochemical MBR. This indicates that the electrochemical MBR was much effective in mitigating polysaccharides-related fouling. Polysaccharides have been reported to induce severer fouling than proteins by a group of researchers^36^,^37^,^38^, and thus the removal of polysaccharides in this study enhanced the filtration performance of the conductive membrane in the electrochemical MBR. One reason in mitigating polysaccharide fouling is the slightly lower polysaccharide concentrations in both SMP and EPS for the electrochemical MBR (2.6 ± 0.9 mg/L for SMP and 63.5 ± 21.4 mg/L for EPS) than the control MBR (2.9 ± 1.4 mg/L for SMP and 71.3 ± 20.6 mg/L for EPS). However, the polysaccharide/protein ratios (poly/pro) in SMP and EPS of the electrochemical MBR were 1.15 and 0.26, which were higher than those in SMP and EPS of the control MBR (see Table S3). Therefore, another reason may be attributed to the fact that the electrostatic repulsive force between membrane and polysaccharides was enhanced in the presence of electrical field. However, the removal of proteins might be impacted by the charge heterogeneity of proteins since they contain negatively charged carboxyl groups and positively charged amine groups^39^,^40^. The other important factor should be ascribed to the reaction between H_2_O_2_ and the foulants. Polysaccharides usually have abundant hydroxyl groups^41^, which can be oxidized to carboxyl groups in the presence of H_2_O_2_ (H_2_O_2_ concentration in the electrochemical MBR was about 0.95 ± 0.21 mg/L as documented earlier). This in turn increases the absolute value of zeta potential of polysaccharides, thus improving the repulsive force between polysaccharides and membranes. The above-mentioned two reasons might explain why the conductive membrane in the electrochemical MBR was effective in mitigating polysaccharides-related fouling. In combination with CLSM analysis, the major mechanism for fouling mitigation can be schematically illustrated in Fig. 7B. Overall, fouling mitigation in the continuous-flow electrochemical MBR should be attributed to the enhanced repulsive force, *in-situ* H_2_O_2_ cleaning (in particular efficient in removing polysaccharides under investigated conditions), and decreased SMP and EPS concentrations as mentioned earlier.

**Figure 8:**
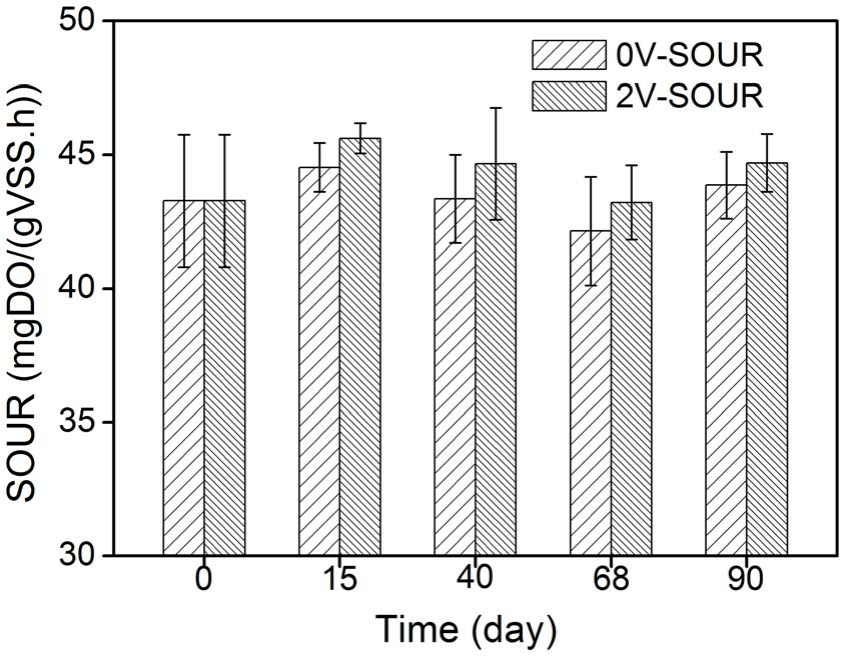
Comparison of SOUR in the MBRs with and without electric field at different operation time. Error bars represent standard deviations of triplicate measurements.

Although this technology demonstrates its feasibility in fouling suppression, one thing that is worth noting is the potential negative impacts of electric field on microbial activity in continuous-flow MBRs. For clarifying this, specific oxygen uptake rates (SOUR) of the activated sludge in the two MBRs were determined, and the results are shown in Fig. 8. Compared with the control MBR, the SOUR of the electrochemical MBR was slightly higher (Fig. 8), indicating that the activity of sludge was enhanced under this electric field intensity of 2 V/cm. Alshawabkeh et al. observed that the optimal electric field benefiting microorganisms was 0.28–1.4 V/cm, and an applied electric field greater than 1.4 V/cm may be harmful for microorganism^42^. The impacts of electric field on sludge properties are also dependent on current density, electrical exposure mode and MLSS concentration^43^. For instance, Bayar and Karagunduz found that the sludge activity was not affected even at the electric field intensity of 5 V/cm^44^. The obtained results in this study suggest that the applied 2 V/cm electric field had no adverse impacts on microbial activity. This is also consistent with the pollutant removal in the electrochemical MBR.

Another aspect that is worth noting is about the chemical scaling in the presence of electric field. In this study, we found insignificant differences of ion contents in membrane foulants at the end of experiments (see Table S4). For instance, the Ca^2+^ contents on the fouled membrane with electricity and the control membrane were 1.52 ± 0.08 g/m^2^–membrane and 1.69 ± 0.09 g/m^2^–membrane (*n* = 3), respectively. There were also insignificant changes for Fe and Mg contents. Nevertheless, the long-term operation of MBRs fed with real municipal wastewater should be investigated.

Another issue of concern is the energy consumption by applying an external electric field for this technology. In this study, current density through anode and cathode under applied electric field of 2 V/cm was continuously recorded, which was about 2.0 mA on average. The specific energy consumption was about 0.038 kW**·**h/m^3^-wastewater for this lab-scale MBR (calculated by Eq.(3)). If membrane area is increased to 1 m^2^ under the same electricity field, the specific energy consumption will be 0.014 kW**·**h/m^3^-wastewater (the detailed calculation procedure is documented in Supporting Information). It is evident that the extra electricity energy consumption for this electrochemical MBR by applying an external electric field is minute compared to a typical energy consumption of 0.6 kW**·**h/m^3^ for wastewater treatment^45^,^46^.

In summary, the results of this study provide evidence for fouling mitigation in MBRs through preparing conductive MF membrane and applying appropriate electric field. The long-term operation of this conductive MF membrane shows good electrochemical properties and distinct anti-fouling ability. The use of 2 V/cm electric field also has no negative impacts on microbial viability. Compared to wastewater treatment costs, the specific energy consumption of exerting electric field is also negligible, demonstrating its strong potential for real applications. Additional studies are needed to optimize membrane preparation, operating conditions (electric field density) and reactor configuration design in order to apply this technology for real wastewater treatment.

## Methods

### Conductive membrane preparation

Commercial grade polyvinylidene fluoride (PVDF) materials were purchased from Shanghai 3F New Material (China). Dimethyl sulphoxide (DMSO) was used as the solvent, and polyethyleneglycole (PEG 600) as the pore-forming additive, which were both purchased from Sinopharm (Shanghai, China). Deionized (DI) water was used throughout the experiment. Membrane casting solution was prepared according to the procedures as documented by Wang et al.^47^. The homogeneous casting solution was coated on stainless steel mesh (pore size 96 μm, thickness 43 μm) assembled on polyester non-woven fabric. The composite membrane was formed after immersion precipitation in non-solvent bath.

### Membrane characterization

Membrane morphologies were observed with scanning electron microscope (SEM) (Model XL-30, Philips, Netherland). Membrane samples were dried at 40°C for 48 h before observation. Membrane pore size and surface porosity were determined by the software (ImageJ, NiH). Roughness was measured using an atomic force microscope (AFM) by the software (NanoScope Analysis). Each reported value was obtained by averaging at least five measurements. Fouled membranes were visualized using confocal laser scanning microscope (CLSM) according to the procedures in our previous publication^48^.

Contact angle (CA) was measured using an optical contact angle measurement system (JC2000D, Yisu Co. Shanghai, China). Five microliters of water was dropped on investigated membrane surface from a micro-syringe with a stainless needle at room temperature (25 ± 1°C). At least seven measurements at random locations were averaged to obtain a CA value for a membrane sample.

Pure water flux (PWF) was determined at a trans-membrane pressure (TMP) of 30 kPa using a dead-end filtration cell (250 mL, Chenyi Co. Shanghai, China). PWF is worked out using Eq. (1):
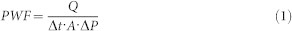
where *Q* is the volume of filtrate collected (L), Δ*t* is the filtration time (h), *A* is the membrane area (m^2^) and Δ*P* is the trans-membrane pressure (kPa).

Samples of conductive MF membrane and control membrane (without steel stainless mesh) with an effective width and length of 4 cm × 32 cm were prepared for tensile strength testing using a CMT4204 microcomputer controlled electronic universal testing machine (Sans Material Testing Corporation, China) at room temperature. A strain rate of 10 mm/min was employed. The reported values were the averages of at least five samples.

Linear sweep voltammetry (LSV) and electrochemical impedance spectroscopy (EIS) were used to electrochemically characterize the conductive MF membrane using an electrochemical workstation (CS350, Corrtest Co., China). LSV was performed using a three-electrode cell, consisting of a working electrode (cathode), an Ag/AgCl reference electrode (0.197 V vs. SHE), and a counter electrode (Pt). The conductive MF membrane (cathode) with surface area of 1.0 cm^2^ was scanned at 1 mV/s from +0.6 V to 0 V (vs. Ag/AgCl). A stainless steel mesh was also measured for comparison. Current density was normalized to the projected surface area of cathodes. EIS of the cathodes was measured in the continuous-flow bioreactor with a three-electrode arrangement, in which the anode was used as the counter electrode at a sinusoidal perturbation of 10 mV amplitude over a frequency range of 10^5^ to 10^−2^ Hz.

### Membrane batch filtration of model foulants

Bovine serum albumin (BSA), sodium alginate (SA), humic acid (HA) and silicon dioxide (SiO_2_) particles, which were purchased from Sigma Aldrich, were used as model foulants to evaluate antifouling performance of the conductive MF membrane with an external electrical field. Properties of the model foulant solutions are shown in Table S2. Zeta potentials and particle sizes were determined by dynamic light scattering (DLS) with a Malvern Zetasizer (Malvern Instruments Limited, UK). pH value of the model foulant solutions was adjusted to 8.5 with 1 mol/L NaOH, and determined by a pH meter (HQ40d, Hach, America).

External electric field was supplied by a DC power (CHI1030C, Jiecheng Co., Shanghai, China). The anodes made of two pieces of carbon cloth were placed at either side of the membrane module with 1 cm distance from the conductive membrane, while the conductive membrane directly served as the cathode. Tests were conducted for three cycles and each lasted for 3.0 h at trans-membrane pressure 3.0 kPa. For eliminating errors brought by fouling, each test used a new membrane module. Relative flux (the ratio of a dynamic flux to the initial membrane flux, *J*/*J*_0_) as a function of time was shown for comparing flux bahaviours with and without electric field.

### Continuous-flow MBR system

In order to evaluate the anti-fouling performance of the conductive MF membrane, two parallel MBRs were used for comparing their filtration behaviours with and without electric field (2 V/cm). A schematic diagram of the MBRs is shown in Fig. S3. Each reactor had an effective volume of 630 mL and was installed with a flat sheet membrane module with an effective area of 48 cm^2^. Copper line was connected to membrane surface with the silver paste conductive adhesive, and the connector was sealed with epoxy to avoid metal corrosion in long-time operation. A perforated Plexiglas tube was mounted below the membrane module to supply oxygen for microbes and to maintain a cross-flow velocity (CFV) along membrane surface.

The inoculum sludge was collected from an anaerobic/anoxic/oxic-MBR, which has been operated for more than 3 years^49^. Mixed liquor suspended solids (MLSS) and mixed liquor volatile suspended solids (MLVSS) concentrations were 6.0 g/L and 3.8 g/L, respectively. After the inoculation, synthetic wastewater was continuously fed into the reactor through a peristaltic pump (Lange Co., Baoding, China). The composition of the synthetic wastewater was as follows: CH_3_COONa 640 mg/L, NH_4_Cl 77 mg/L, Na_2_HPO_4_ 27 mg/L, CaCl_2_ 11.5 mg/L, MgSO_4_ 12 mg/L, and 10 mL of the trace element solution, which was also used by others^13^,^50^. The effluent was extracted through the flat-sheet membrane using a peristaltic pump (Lange Co., Baoding, China). Two stages were carried out by changing the specific aeration demands (SAD_m_), i.e., SAD_m_ of 100 m^3^/(m^2^·h) at stage 1 and 150 m^3^/(m^2^·h) at stage 2. Membrane flux was maintained constant as 25 L/(m^2^·h) during the experiment. Sludge retention time (SRT) was 30 d by daily discharging excess activated sludge. TMP was monitored by a pressure gauge to indicate the anti-fouling performance of membranes. Current through anode and cathode was monitored by a DC power (CHI1030C, Jiecheng Co. Shanghai, China). MBRs were operated at room temperature (25 ± 1°C).

The electricity energy consumed in the electrochemical MBR can be worked out through Eq. (2).
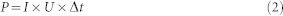
where *P* is the consumed energy (W**·**h), *I* is the current density monitored by the electrochemical workstation (A), *U* is the voltage (V) and Δ*t* is the operation time (h).

The specific energy consumption per m^3^ treated wastewater (*E*) can be calculated according to Eq. (3).
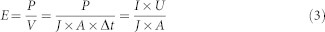
where *V* is the permeate volume (m^3^), *J* is the membrane flux (m^3^/(m^2^ h)) and *A* is the membrane surface area (m^2^).

### Analytical methods

Centrifugation-ultrasonication method was used to extract SMP and bound EPS from sludge samples^51^. Protein and humic acid concentrations in the SMP and bound EPS samples were measured by a corrected Lowry method^52^ and carbohydrates were determined using the phenol-sulfuric acid method^53^. Zeta potentials of SMP and EPS were determined by dynamic light scattering (DLS) with a Malvern Zetasizer (Malvern Instruments Limited, UK).

H_2_O_2_ concentration produced around the cathode was spectrophotometrically determined using the vanadate method^12^,^54^. Chemical oxygen demand (COD) was detected using Hach reagents (HR, Hach, America). Ammonium (NH_3_-N) was assayed using APHA standard methods^55^.

Specific oxygen uptake rates (SOUR) of the two MBRs' sludge were analyzed at 25 ± 1°C. The synthetic wastewater as mentioned earlier was used as the nutrient media for SOUR determination. Dissolved oxygen (DO) was monitored by a DO meter (HQ40d, Hach, America).

To determine ion concentrations on the membrane surface, samples with surface area of 4.0 cm^2^ (1 cm × 4 cm), which were cut from membranes in two MBRs at the end of operation, were soaked in 10 ml hydrochloric acid of 1 mol/L for 12 h. Then the solution was filtered by a 0.22 μm membrane and the filtrate was determined using an inductive coupled plasma emission spectrometer (Agilent 720ES, USA). The ion contents were converted to specific weight per unit membrane (g/m^2^-membrane).

## Author Contributions

J.H. carried out the experiments, analyzed the data, and wrote the main manuscript; Z.W.W. designed the experiments, analyzed the data, and wrote the manuscript; J.Y.Z. and X.R.Z. carried out experiments; J.X.M. and Z.C.W. analyzed the data and discussed the scientific idea.

## Supplementary Material

Supplementary Information Supplementary information
