# Supplementary material for: A novel composite conductive microfiltration membrane and its anti-fouling performance with an external electric field in membrane bioreactors
Source: Sci Rep. 2015 Mar 18;5:9268. doi: 10.1038/srep09268 (PMC4363883; doi:10.1038/srep09268)
Supplement: Supplementary Information [file srep09268-s1.pdf]

Supplementary Materials

A novel composite conductive microfiltration membrane and its anti-fouling performance with an external electric field in membrane bioreactors

Jian Huang, Zhiwei Wang*, Junyao Zhang, Xingran Zhang, Jinxing Ma, Zhichao Wu

State Key Laboratory of Pollution Control and Resource Reuse, College of Environmental Science and Engineering, Tongji University, Siping Road 1239, Shanghai 200092, P.R. China

This supplementary material contains 4 tables (Table S1, Table S2, Table S3 and Table S4), 3 figures (Fig. S1, Fig. S2 and Fig. S3) and information regarding energy calculation.

*Corresponding author. Tel./fax: +86-21-65980400. E-mail address: zwwang@tongji.edu.cn

Table S1. Membrane properties of the conductive MF membrane. Values are expressed as average ± standard deviations; *n*=3 for PWF; *n*=7 for contact angle; *n*=5 for roughness; *n*=40 for pore size.

Items	Value
Pure water flux (L/(m^2·h·kPa))	66.3 ± 4.6
Contact angle (^{o})	69.8 ± 4.3
Root mean-square roughness (*R*q) (nm)	71.9 ± 2.6
Average roughness (*R*a) (nm)	49.4 ± 5.1
Pore size (μm)	0.062 ± 0.024

Table S2. Properties of the model foulant solutions (*n*=6). The particle size of silicon dioxide is reported by the manufacturer.

Model foulants	Concentration (mg/L)	Zeta Potential (mV)	Particle Size (nm)
Bovine serum albumin	50	-43.3 ± 2.3	547.5 ± 63.2
Sodium alginate	50	-38.1 ± 1.8	242.3 ± 42.3
Humic acid	50	-40.9 ± 3.8	314.7 ± 21.5
Silicon dioxide particle	1000	-47.8 ± 2.4	2000

Table S3 Polysaccharides and proteins concentrations in SMP and EPS in the electrochemical MBR and the control MBR.

Foulants	0 V/cm			2 V/cm		
	Polysaccharides (mg/L)	Proteins (mg/L)	Poly/pro	Polysaccharides (mg/L)	Proteins (mg/L)	Poly/pro
SMP	2.9 ± 1.4	3.4±2.6	0.85	2.6 ± 0.9	2.3±1.9	1.15
EPS	71.3 ± 20.6	292.6±66.1	0.24	63.5 ± 21.4	248.4±56.3	0.26

Table S4. Three major divalent/multivalent ion contents on fouled membranes (n=3).

Ions	Quantity (g/m^2-membrane)	
	0 V/cm	2V/cm
Ca^{2+}	1.69 ± 0.09	1.52 ± 0.08
Fe^{n+}	0.05 ± 0.003	0.03 ±0.001
Mg^{2+}	0.21 ± 0.011	0.21 ±0.010

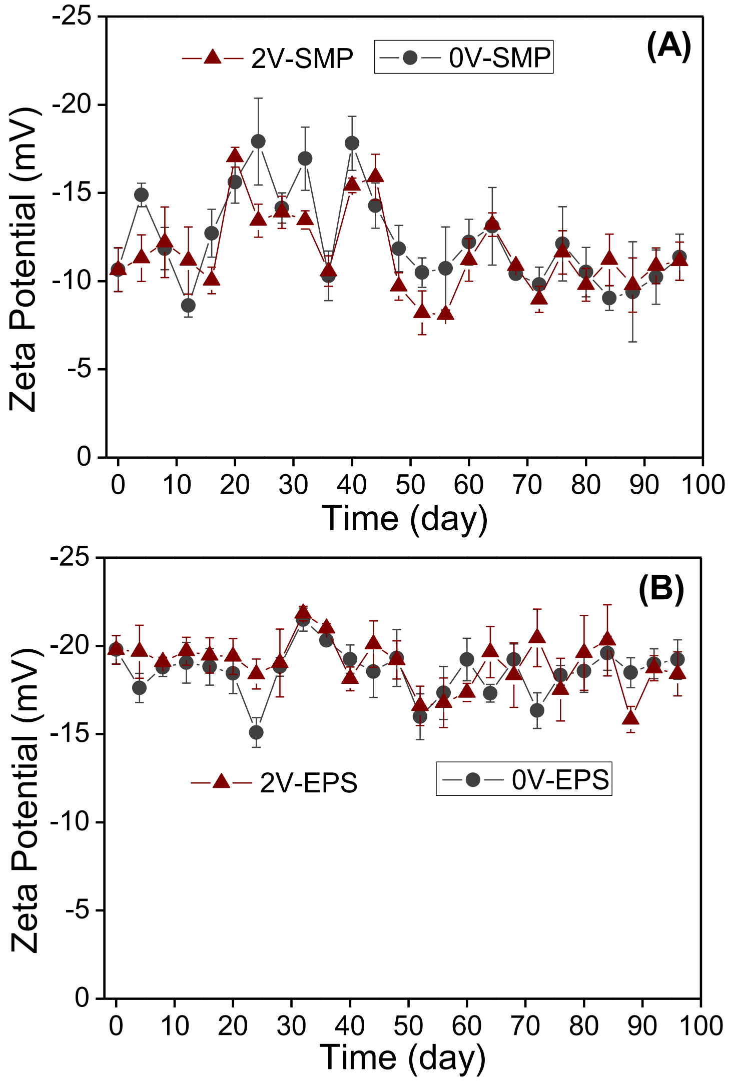

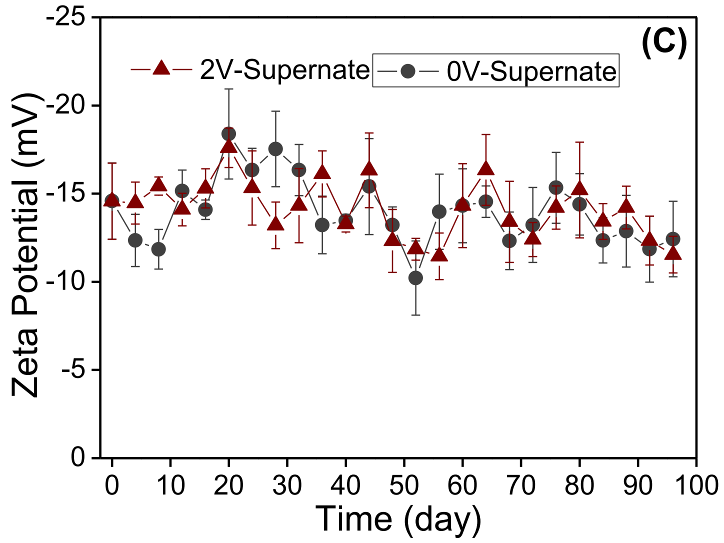

Fig. S1 Zeta potentials of (A) SMP, (B) EPS and (C) activated sludge supernatant for the two MBR systems

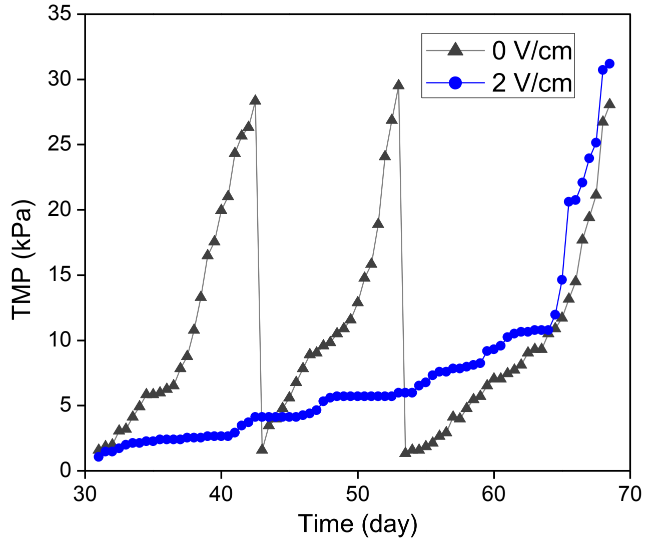

Fig. S2. Comparison of TMP evolution between two membrane modules in one reactor.

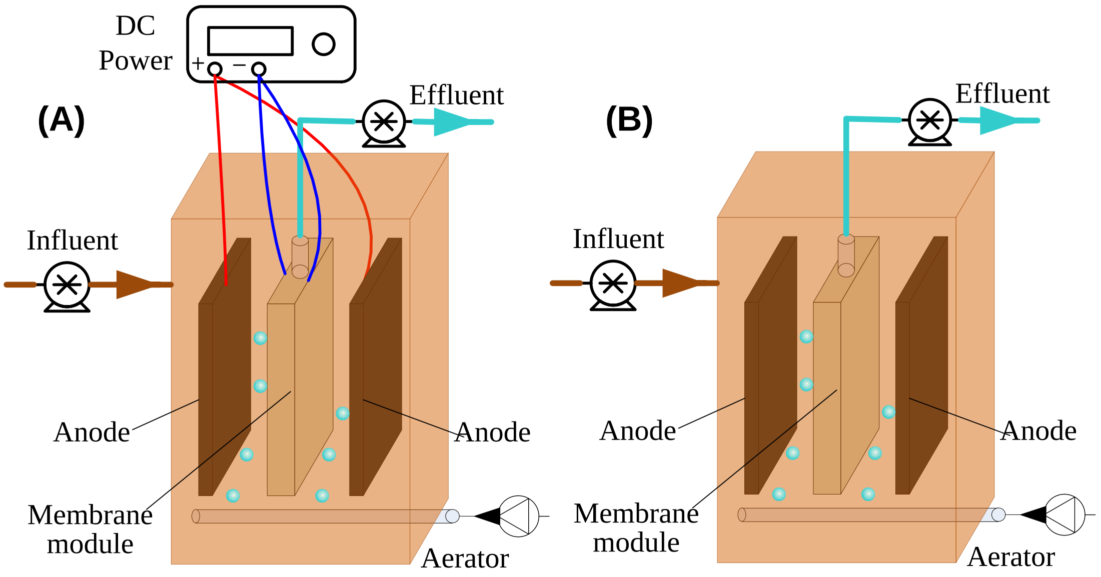

Fig. S3. Schematic diagram of MBR systems. (A) Electrochemical MBR with an external electric field, (B) control MBR without an external electric field.

Energy consumption calculation

The electricity energy consumed in the electrochemical MBR can be worked out according to Eq. (S1).

$$P = I \times U \times \Delta t \qquad \text{(S1)}$$

where *P* is the consumed energy (W·h), *I* is the current density monitored by the electrochemical workstation (A), *U* is the voltage (V) and Δt is the operation time (h).

The applied voltage is 2 V and the average current is about 2.0 mA, and thus the consumed energy for 1 hour is 4.0×10^{-6} kW·h. In lab-scale, the MBR was operated under a flux of 25 L/(m²·h) with an effective membrane area of 4.2×10^{-3} m². After the MBR was operated for 1 hour, the volume of treated water was 1.05×10^{-4} m³. Then we can calculate that the specific energy consumption per m³ treated water is 0.038 kW·h/m³ according to Eq. (S2).

$$E = \frac{P}{V} = \frac{P}{J \times A \times \Delta t} = \frac{I \times U}{J \times A} \qquad \text{(S2)}$$

where *V* is the permeate volume (m³), *J* is the membrane flux (m³/(m² h)) and *A* is the membrane surface area (m²).

However, for practical applications, the membrane area will be increased, leading to an increase of produced water volume and a decrease of electrical resistance of the system. In order to calculate the energy consumption, we further determined the current with the membrane area of 1.0 m^2 under 2 V/cm in a pilot-scale reactor with similar parameters to the lab-scale one, and the value was about 180 mA on average. The volume of treated water is 2.5×10^{-2} m^3 for 1 hour when the membrane area is increased to 1.0 m^2, and the specific energy consumption is 1.4×10^{-2} kW·h/m^3 according to Eq. (S2).
